# Knockdown of Bardet-Biedl Syndrome Gene *BBS9/PTHB1* Leads to Cilia Defects

**DOI:** 10.1371/journal.pone.0034389

**Published:** 2012-03-29

**Authors:** Shobi Veleri, Kevin Bishop, Damian E. Dalle Nogare, Milton A. English, Trevor J. Foskett, Ajay Chitnis, Raman Sood, Paul Liu, Anand Swaroop

**Affiliations:** 1 Neurobiology-Neurodegeneration and Repair Laboratory (N-NRL), National Eye Institute, National Institutes of Health, Bethesda, Maryland, United States of America; 2 National Human Genome Research Institute, National Institutes of Health, Bethesda, Maryland, United States of America; 3 National Institute of Child Health and Human Development, National Institutes of Health, Bethesda, Maryland, United States of America; Instituto de Medicina Molecular, Portugal

## Abstract

Bardet-Biedl Syndrome (BBS, MIM#209900) is a genetically heterogeneous disorder with pleiotropic phenotypes that include retinopathy, mental retardation, obesity and renal abnormalities. Of the 15 genes identified so far, seven encode core proteins that form a stable complex called BBSome, which is implicated in trafficking of proteins to cilia. Though BBS9 (also known as PTHB1) is reportedly a component of BBSome, its direct function has not yet been elucidated. Using zebrafish as a model, we show that knockdown of *bbs9* with specific antisense morpholinos leads to developmental abnormalities in retina and brain including hydrocephaly that are consistent with the core phenotypes observed in syndromic ciliopathies. Knockdown of *bbs9* also causes reduced number and length of cilia in Kupffer's vesicle. We also demonstrate that an orthologous human *BBS9* mRNA, but not one carrying a missense mutation identified in BBS patients, can rescue the *bbs9* morphant phenotype. Consistent with these findings, knockdown of *Bbs9* in mouse IMCD3 cells results in the absence of cilia. Our studies suggest a key conserved role of BBS9 in biogenesis and/or function of cilia in zebrafish and mammals.

## Introduction

The cilium is a specialized organelle projecting from plasma membrane of most polarized cell types in vertebrates [1], [2]. The cilium develops from the basal body that is in turn derived from the mother centriole and participates in many fundamental signaling pathways, including those associated with sonic hedgehog [3], Wnt [4] and planar cell polarity (PCP) [5]. The vertebrate cilium is estimated to contain over 1000 proteins for its structural and/or functional integrity [6] (http://www.ciliaproteome.org). Defects in cilia biogenesis are associated with pleiotropic syndromic phenotypes, collectively referred as ciliopathies; these include Bardet-Biedl Syndrome (BBS), Meckel-Gruber Syndrome (MKS), Joubert Syndrome (JBTS), and Nephronophthesis (NPHP) [7].

Bardet-Biedl syndrome (MIM#209900) is typically an autosomal recessive disorder that exhibits variable expressivity and phenotypes including retinopathy, mental retardation, obesity, polydactyly, and renal abnormalities [8], [9]. Mutations in fifteen genes are reported to account for 80% of the BBS cases [9], [10]; a few of these are also associated with the pathogenesis of related ciliopathies. Despite tremendous genetic heterogeneity, all BBS proteins are localized to centrosome, basal body or the ciliary transition zone [9], [11], [12], [13], [14], [15]. Investigations using mouse and zebrafish models have demonstrated the cilia-associated functions of BBS8, BBS4 and other BBS proteins [5], [13], [16], [17]. Similarities in clinical phenotypes and cellular localization have suggested interaction(s) among different BBS proteins and their participation in cilia biogenesis, signaling or transport. Identification of two multiprotein complexes that include BBS proteins has provided key biochemical and functional insights into cilia biology and disease. BBSome, a stable complex of seven core BBS proteins (BBS1, BBS2, BBS4, BBS5, BBS7, BBS8, BBS9) is implicated in cilia trafficking and biogenesis [18], whereas the chaperonin complex (comprising of BBS6, BBS10, and BBS12) seems to mediate the assembly of BBSome [19].

*BBS9* (also called *PTHB1*) was originally identified by differential display analysis as a gene (*B1*) down regulated by parathyroid hormone (PTH) in an osteoblastic cell line [20]. Multiple variant isoforms of *PTHB1* are expressed in different tissues, and the gene is interrupted in a translocation associated with Wilms' Tumor 5 [21]. More recently, haplotypes in the region of *PTHB1* have been associated with the pathogenesis of premature ovarian failure, a complex multifactorial disease that causes female infertility [22]. Independent genetic studies, involving comparative mapping and gene expression analysis, led to the identification of *PTHB1* as a novel BBS gene – *BBS9* [23]. Mutations in *BBS9* account for 6% of BBS mutations [9].

Though BBS9 protein is shown to be a part of BBSome core [18], its precise physiological function is not delineated, and the mechanism of disease pathogenesis caused by *BBS9* mutations is poorly understood [23]. The zebrafish (*Danio rerio*) has been used as an excellent system to model human diseases, especially those involving ciliary protein functions, by knockdown using morpholino (MO) technology [16]. Knockdown of many cilia genes in zebrafish are reported to cause developmental abnormalities in the eye, brain and somites [24], [25], [26]. Here we report that *bbs9* knockdown results in ciliogenesis defects in zebrafish and in mouse IMCD3 cells.

## Results

### A *BBS9* ortholog is expressed in the zebrafish during development

**Figure 1:**
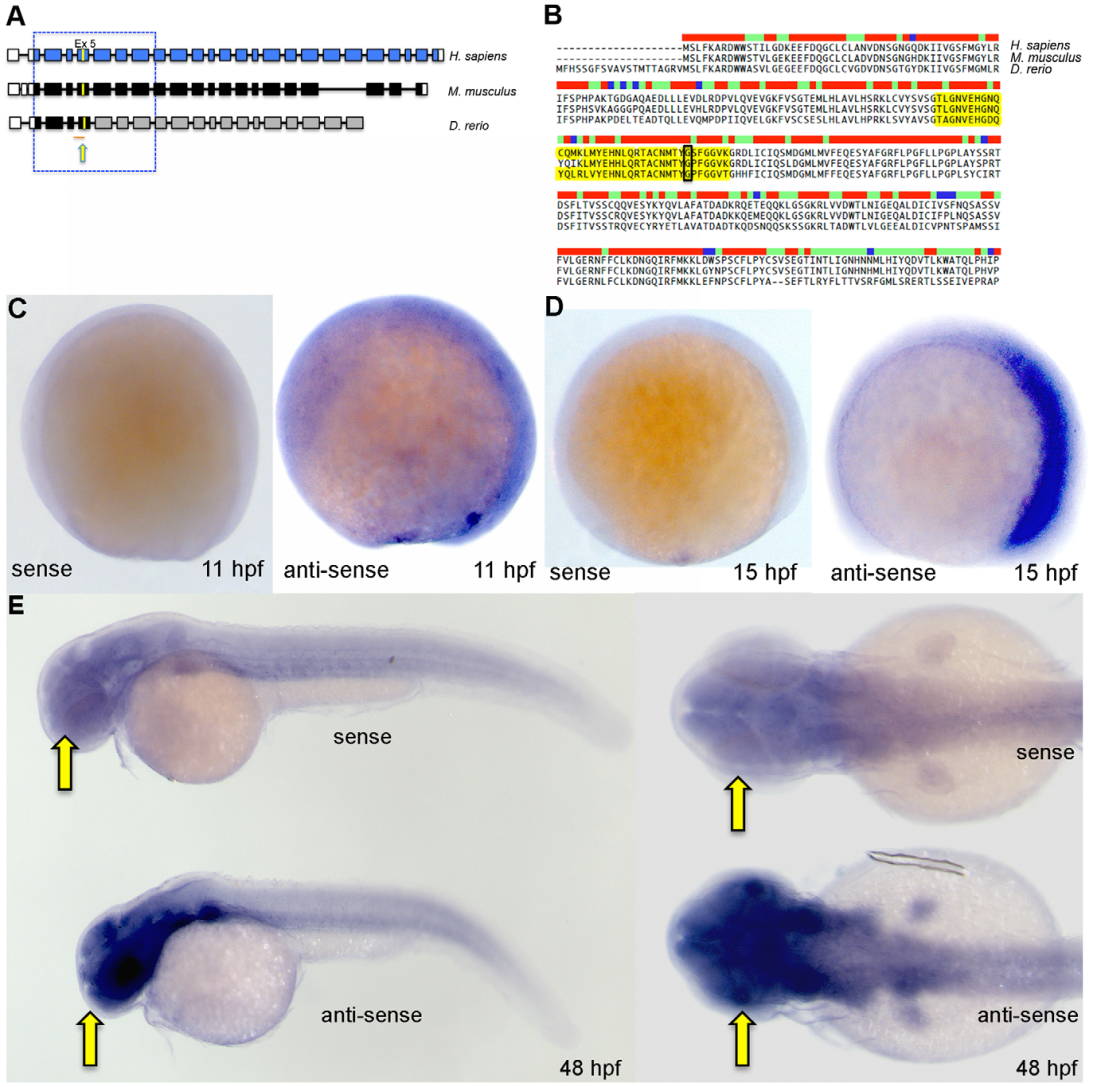
Zebrafish *bbs9* gene: structural comparison and expression pattern. (**A**) A comparison of *BBS9* exon:intron structure between human (*H. sapiens*, top blue), mouse (*M. musculus*, middle black) and zebrafish (*D. rerio*, bottom gray/black). The filled and open boxes indicate coding exons and UTRs, respectively. The blue and black boxes represent validated exons. The gray boxes represent exons present in provisional sequence XM_002664792.1. Exons 2 to 8 are highly conserved across species (boxed area within hatched square). The yellow arrow points to yellow mark on exon 5, which represents the missense mutation G→A (p.G141R) in human BBS9 protein. Under the zebrafish *bbs9* transcript, the red line represents *bbs9*-spMO targeting site at intron4:exon5 boundary. (**B**) The protein sequence alignment (clustalW) between human (NP_940820.1), mouse (NP_848502.1) and predicted zebrafish BBS9 (904 amino acids). Exon 5 is highlighted (yellow), and the position of missense mutation (p.G141R) in human is highlighted by a black rectangle. The bar coding on top of the sequences represents degree of conservation (red and blue represent maximum and minimum conservation, respectively). (**C, D**) *In situ* hybridization analysis at 11 hpf and 15 hpf. Left and right panels represent the sense and anti-sense probes generated from *bbs9* cDNA. (**E**) *In situ* hybridization analysis at 48 hpf. Expression of *bbs9* in the eye, brain and somites gives a strong signal with the anti-sense probe compared to the background signal from the sense probe. Compare the strong signal in the head regions (arrows). Left and right panels represent lateral and dorsal views, respectively.

In order to test the *in vivo* function of *bbs9* using zebrafish, we first identified the zebrafish ortholog of human *BBS9* gene (NM_198428) using available resources from GenBank (XM_002664792.1), Zfin (AL845419) and GENESCAN program (http://genes.mit.edu/GENESCAN.html). The predicted transcript codes for a protein of 904 amino acids that shows 63% identity and 79% similarity with human BBS9 protein (Fig. 1A, B). A multi-species comparison of BBS9 protein sequences revealed high level of conservation from exons 2 to 8 among human, mouse and zebrafish (Fig. 1B). Furthermore, the region of zebrafish *bbs9* on chromosome 16 is syntenic with the human *BBS9* locus on chromosome 7. Currently, there is no evidence for additional copies of *bbs9* in zebrafish genome.

To analyze the expression of *bbs9* during development, we generated a partial cDNA spanning exons 2–5 by RT-PCR from 72 hour post-fertilization (hpf) embryos. *In situ* hybridization with antisense mRNA probe generated from this cDNA to 11 hpf zebrafish embryos showed almost ubiquitous expression (Fig. 1C); however, by 15 hpf, *bbs9* expression became restricted to the anterior portion of the embryo (Fig. 1D). At 48 hpf, *bbs9* transcripts were expressed in high levels in eyes and brain, while the somites displayed low level expression (Fig. 1E).

### Validation of *bbs9* morpholinos

**Figure 2:**
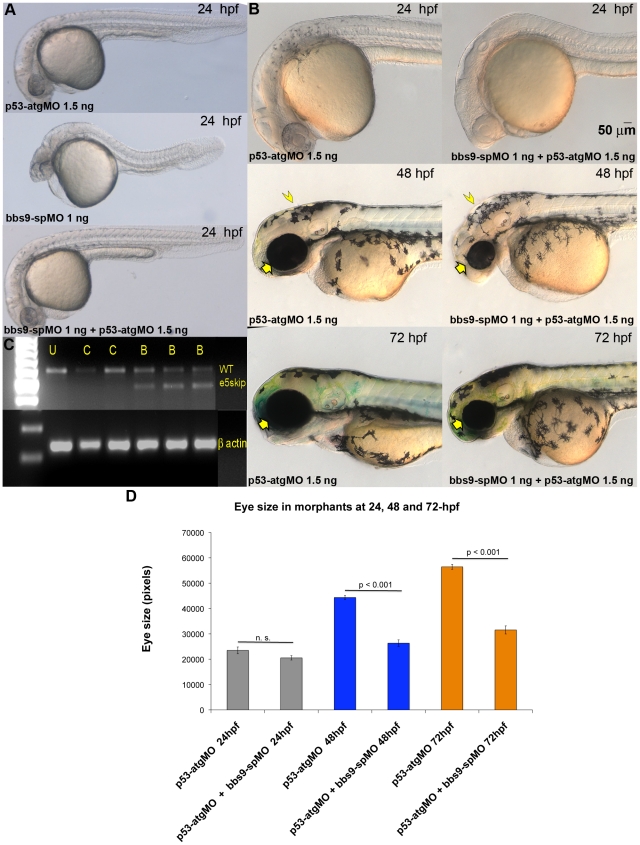
Exon 5-targeted *bbs9* splice morpholino affects eye development independent of p53 pathway. (**A**) At 24 hpf, the *p53*-atgMO (1.5 ng) alone injection did not elicit a phenotype. The *bbs9*-spMO (1 ng) injection alone caused developmental defects in the eye, brain and tail of morphants. However, co-injection of *p53*-atgMO reduced the defects seen by the *bbs9*-spMO injection alone, though mild eye defect remained the tail becomes normal (bottom panel). (**B**) Higher magnification of morphants' head region. Top, middle and bottom rows are 24-, 48- and 72-hpf, respectively. Left and right column of panels are *p53*-atgMO without and with *bbs9*-spMO, respectively. At 48 hpf the effect of *bbs9*-spMO injection on eye size visible (compare the arrows). The *bbs9*-spMO injection also resulted in hydrocephalous (compare the arrow heads). The defects seen at 48 hpf are weaker at 72 hpf. (**C**) The gel photograph of RT-PCR showing exon-skipping by *bbs9*-spMO. mRNA isolated from individual embryos was used for RT-PCR. U, C (4 and 6 ng) and B (1, 4, 6 ng) represent un-injected, control, and *bbs9*-spMO, respectively. Splice blocking gave an additional smaller (marked e5skip) band along with the original WT band. The bottom panel shows β-actin control for respective samples. (**D**) Quantification of the effect of morpholino(s) injection on eye size. X-axis shows the morpholinos used and time (hpf) of scoring. Y-axis shows eye size in pixels (mean ± SEM).

As the only known missense mutation in BBS9 patients is in exon 5 and another frame-shift mutation in intron 4 affected exon 5 [23], we designed an exon-skipping morpholino (*bbs9*-spMO) targeting the intron 4:exon 5 boundary of zebrafish *bbs9* gene (Fig. 1A, red underline). To examine whether *bbs9*-spMO indeed blocked splicing, we performed RT-PCR analysis using RNA from control-MO and *bbs9*-spMO injected morphants (Fig. 2C). A single 575 bp wild type RT-PCR product was observed in control-MO injected morphants, whereas in *bbs9*-spMO injected morphants a shorter product (461 bp, presumably generated by exon 5 skipping, termed e5skip) was detected in addition to the 575 bp band (Fig. 2C). The presence of two bands in the latter indicated an incomplete effect of *bbs9*-spMO in the morphants.

### *bbs9* knockdown causes developmental defects in zebrafish

Microinjection of *bbs9*-spMO into wild type embryos resulted in severe morphogenesis defects (Fig. 2). At 48 hpf, embryos injected with 1 ng *bbs9*-spMO showed a striking defect in the eye and a conspicuous hydrocephaly in most cases (Fig. 2B, middle panels). In initial studies with the *bbs9*-spMO extensive cell death was seen at 24 hpf and there was dose dependent malformation of the trunk and tail (Fig. 2A). As some morpholinos are known to produce p53 dependent cell death [27], we co-injected *p53*-atgMO to determine if this cell death is responsible for a subset of the observed phenotypes. In embryos co-injected with 1 ng *bbs9*-spMO and 1.5 ng *p53*-atgMO, trunk and tail malformations was suppressed (Fig. 2A, bottom panel) compared to embryos injected with *bbs9*-spMO only (Fig. 2A, middle panel). However, reduction in the size of the eye and hydrocephaly was consistently observed at 48 hpf in embryos co-injected with *p53*-atgMO (Fig. 2B, middle row, right panel), and a statistically significant reduction in eye size was seen at 48 and 72 hpf (Fig. 2D), confirming that these changes are *bona fide* effects of reduced bbs9 function. The *bbs9*-spMO morphant phenotype observed with *p53*-atgMO co-injection is reminiscent of what has been reported for BBS patients with clinical manifestations in multiple organs - including eye and brain (see MIM ID #209900).

To confirm the results obtained using the splice blocking *bbs9* morpholino, we designed a *bbs9*-atgMO to target the first translational initiation site, *aug*, in the predicted zebrafish *bbs9* open reading frame (Fig. S1). After 48 hpf, *bbs9*-atgMO injected morphants, co-injected with *p53*-atgMO, displayed a similar reduction in eye size (data not shown) though, overall, *bbs9*-atgMO injected morphants displayed a slightly milder phenotype, with no hydrocephaly.

### Human *BBS9* mRNA rescues *bbs9* knockdown phenotype in the zebrafish

**Figure 3:**
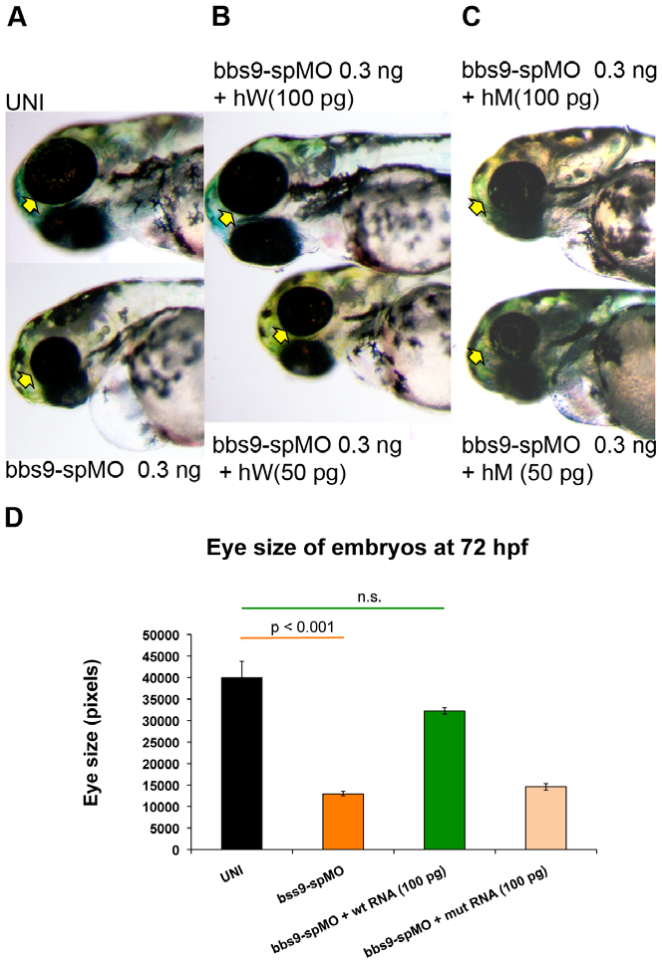
Human mRNA rescues zebrafish *bbs9*-spMO phenotype. h*W* and h*M* represent wild type and mutant human mRNA, respectively. The arrows indicate eye phenotype. (**A**) The uninjected control (top) and *bbs9*-spMO alone injected (bottom) zebrafish at 72 hpf. (**B**) Rescue of *bbs9*-spMO eye phenotype by hW 100 pg (top), but not by lower dose of 50 pg (bottom). (**C**) The *bbs9*-spMO phenotype is not rescued by h*M* as the eye defect remains in the morphants. (**D**) The quantification of embryos' eye size at 72 hpf in rescue experiment using human mRNAs co-injected with *bbs9*-spMO. X-axis shows category of embryos scored. Y-axis shows the eye size in pixels. Data are presented as mean ± SEM. Statistically significant and non-significant observations are indicated with p value and n.s., respectively.

To confirm the specificity of the *bbs9*-spMO morphant phenotype, we asked whether wild type human *BBS9* mRNA could rescue the *bbs9*-spMO injected morphants. Co-injection of 0.3 ng *bbs9*-spMO along with wild type human *BBS9* mRNA rescued the morphant phenotype in a dose-dependent manner (Fig. 3). The *bbs9*-spMO injection alone resulted in morphants with reduced eye size. Co-injection of *bbs9*-spMO with 100 pg of wild-type human mRNA significantly improved the eye size (Fig. 3B, D). Analysis of RNA from the rescued zebrafish revealed an effective splice blocking of zebrafish *bbs9* transcript (data not shown), suggesting that the phenotypic rescue was indeed by the human mRNA.

A comparable phenotype produced by exon 5 mutation in a *BBS9* patient and by the *bbs9*-spMO in zebrafish prompted us to evaluate whether *BBS9* mRNA carrying the exon 5 missense mutation (amino acid change, G141R) [23] could complement the abnormal morphant phenotypes. As predicted, the co-injection of 100 pg of missense mutant mRNA with *bbs9*-spMO failed to rescue the defects in morphants (Fig. 3C, D). Our data further suggest that human and zebrafish BBS9 proteins are highly conserved at the functional level.

### *bbs9* is required for photoreceptor and brain development

**Figure 4:**
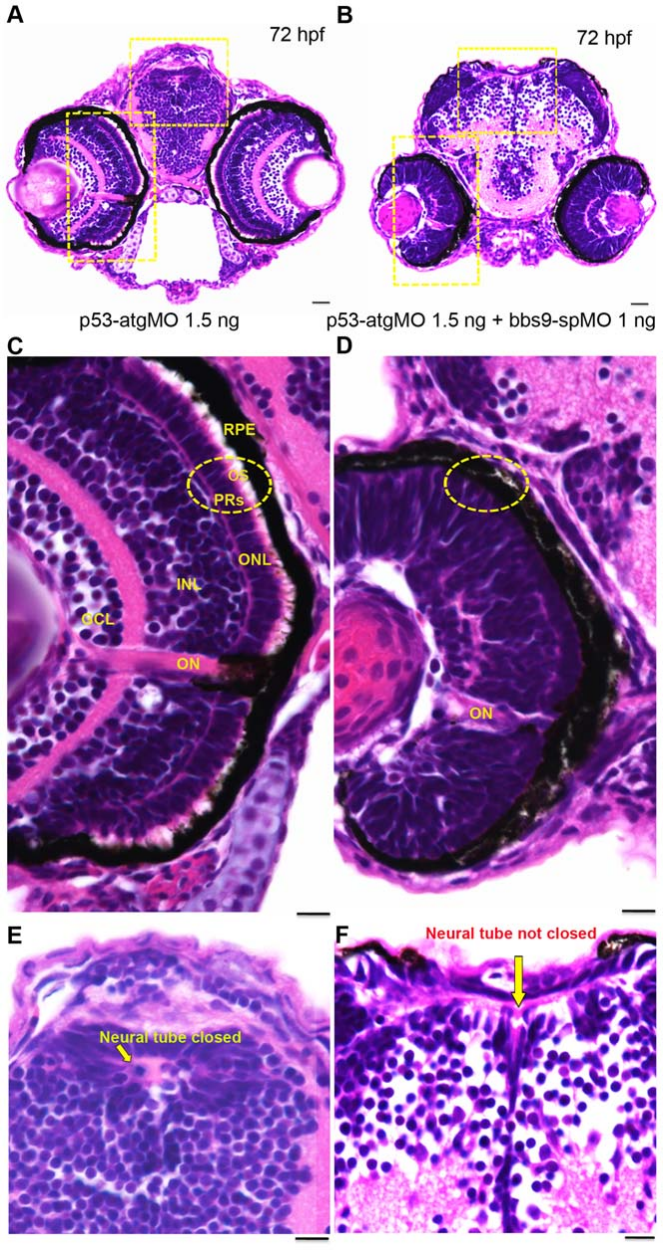
*bbs9*-spMO morphant shows defects in retina lamination and neural tube closure. Zebrafish head sections (72 hfp) stained with H& E. (**A**) The morphant injected with *p53*-atgMO (1.5 ng) shows normal retinal lamination and neural tube (areas highlighted with hatched yellow rectangles). (**B**) The morphant co-injected with *bbs9*-spMO (1 ng) and *p53*-atgMO (1.5 ng) shows lack of retinal lamination and incomplete closure of neural tube (areas highlighted with hatched yellow rectangles). (**C**) The morphant retina in higher magnification - area boxed in ‘**A**’. The retina shows all the 5 layers: Retinal pigment epithelium (RPE) abutting close to photoreceptors (PRs). The PRs (hatched circle) have visible outer segments (OS). Next to PRs are the outer nuclear layer (ONL) followed by an intact inner nuclear layer (INL) and the ganglion cell layer (GCL). The optical nerve (ON) is used as a reference point. (**D**) The *bbs9*-spMO injected morphant retina in higher magnification - area boxed in ‘**B**’. The retina shows no clear lamination and it lacks photoreceptor outer segments (hatched circle). (**E**) The morphant neural tube in higher magnification from ‘A’ shows normal closure (arrow). (**F**) The *bbs9*-spMO injected morphant neural tube in higher magnification from ‘**B**’ shows incomplete closure of neural tube (arrow). The scale bars indicate 100 µm.

*bbs9*-spMO morphants displayed developmental defects in eye and brain, and often with hydrocephaly. These are reminiscent of the clinical features reported in BBS patients [23], [28], [29]. Histological analysis of the morphants' eyes revealed a dose dependent effect of *bbs9*-spMO on photoreceptors compared to uninjected or the control-MO injected embryos (data not shown). To examine whether the eye and brain defects are caused by widespread cell death, we co-injected *p53*-atgMO (1.5 ng) with *bbs9*-spMO (1 ng). At 72 hpf, *p53*-atgMO morphant showed a normal eye and brain development (Fig. 4A), whereas morphants co-injected with *bbs9*-spMO and *p53*-atgMO showed severely malformed eye and brain (Fig. 4B). Our results demonstrate that the eye and brain defects are indeed *bona fide* effects of *bbs9* knockdown.

In *p53*-atgMO, the retina displayed proper lamination with all five layers (Fig. 4, C). The photoreceptors' outer segments were clearly visible (Fig. 4C, circle) abutting the retinal pigment epithelium (RPE). In contrast, co-injection of *bbs9*-spMO caused altered retinal layer stratification apparently forming an amalgam (Fig. 4D). The photoreceptor layer was indistinguishable from RPE, which often displayed denudation from the inner retinal layer due to lack of photoreceptor outer segments (Fig. 4D, circle). The morphants did not develop a full size eye as in the controls (Fig. 4A, B). Absence of properly formed photoreceptors, especially without defined outer segments, argues for compromised cilia function in the *bbs9*-spMO morphants.

We then examined the morphant brain anatomy because of the hydrocephaly, a known sign of ciliary abnormality in the ventricles [26], [28]. Histological analysis of the morphants revealed an effect of *bbs9*-spMO on brain structure. At 72 hpf, the *p53*-atgMO morphants showed normal closed neural tube (Fig. 4A, E), whereas the addition of *bbs9*-spMO resulted in failure of complete neural tube closure (Fig. 4B, F, arrow). Ciliary dysfunction is one of the reasons for incomplete neural tube closure as seen in some of the ciliary mutants [30].

**Figure 5:**
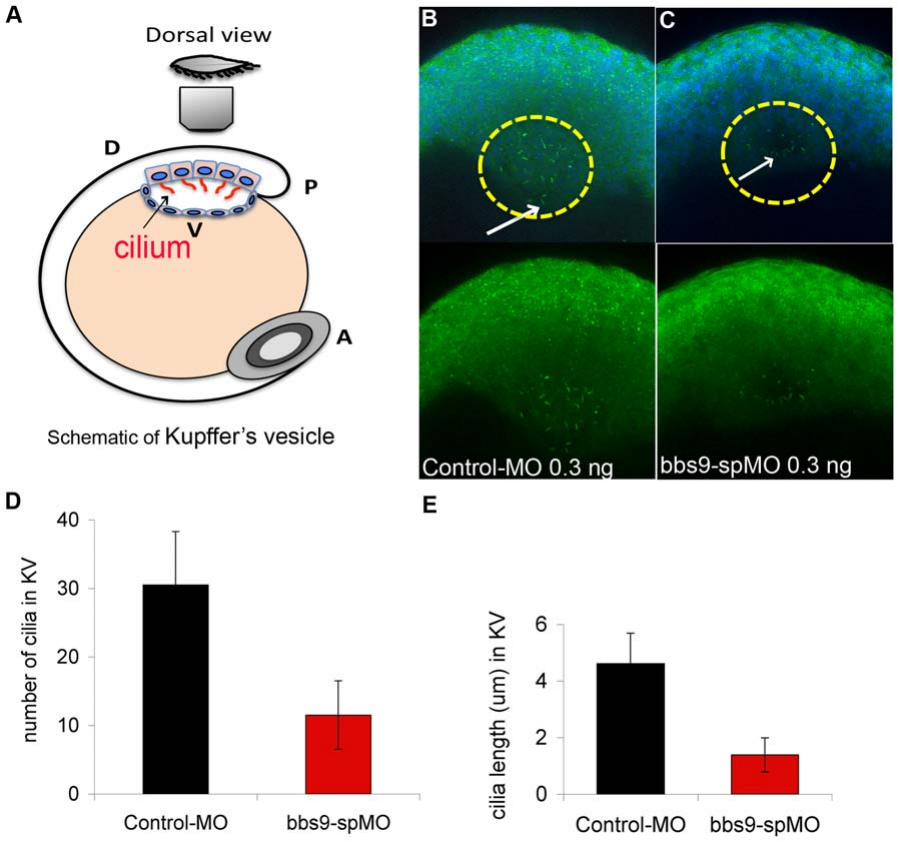
Knockdown of *bbs9* affects cilia in Kupffer's vesicle. (**A**) Schematic view of Kupffer's vesicle (KV) in zebrafish embryo at 12 hpf. A, P, D and V indicate anterior, posterior, dorsal and ventral sides, respectively. (**B**) Morphant injected with control-MO (0.3 ng). (**C**) Morphant injected with *bbs9*-spMO (0.3 ng). In the morphants, KV cilia were visualized by staining with both anti-α-tubulin and anti-γ-tubulin (green), between 10–13 hpf. In ‘**B**’, cilia are more in number and are longer (cf. white arrows) than in ‘**C**’. In B and C, upper panels show the nuclei visualized with DAPI. (**D**) and (**E**) show quantification of KV cilia number and length, respectively. The Y-axis represents the mean ± SEM. The X-axis represents the indicated category of morphants analyzed.

The preceding data prompted us to evaluate whether *bbs9* knockdown affected the cilia in Kupffer's vesicle (KV; Fig. 5A), a structure often afflicted by ciliary dysfunction. We stained morphant (injected with either control-MO or *bbs9*-spMO 0.3 ng) KV cilia with antibodies against acetylated alphaα-tubulin and gammaγ-tubulin. The number of cilia was reduced in *bbs9*-spMO injected morphants compared to control-MO injected morphants (Fig. 5B, C). In *bbs9*-spMO injected morphants, the cilia were less in number and of shorter length compared to the cilia in control-MO injected morphants (Fig. 5D, E). These data further show that cilia biogenesis is compromised in *bbs9*-spMO morphants.

### BBS9 participates in cilia biogenesis in IMCD3 cells

**Figure 6:**
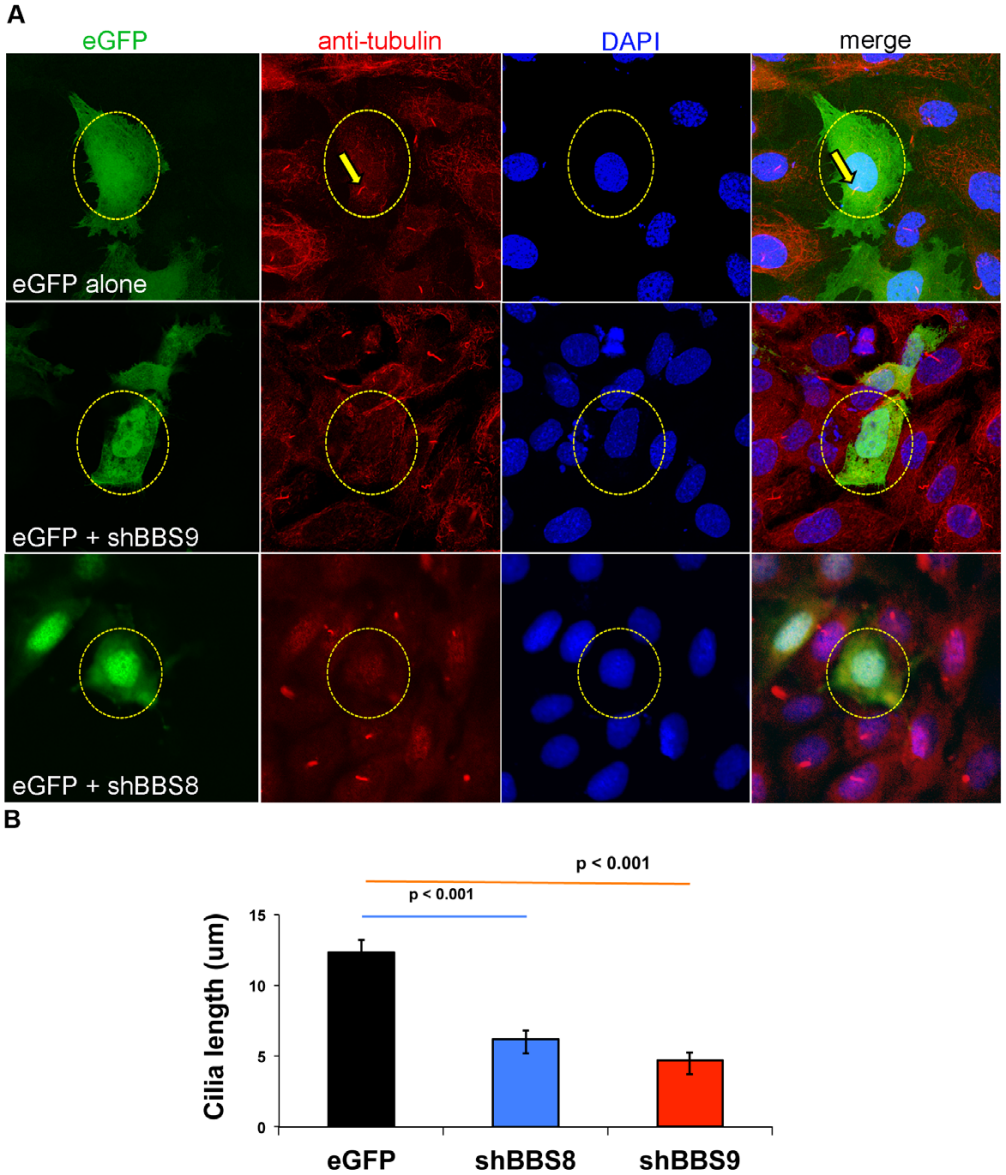
Knockdown of *Bbs9* affects ciliogenesis in IMCD3 cells. (**A**) *Bbs8* and *Bbs9* shRNA transfection in IMCD3 cells. The top row shows eGFP control transfection, whereas middle and bottom rows represent eGFP co-transfected with shRNA against *Bbs9* or *Bbs8*, respectively. The nuclei are visualized with DAPI (blue). Transfection is visualized with eGFP (green). Cilia are visualized with both anti-alpha-tubulin and gamma-tubulin (red). shRNA transfected cells (green) have no cilia (red) - highlighted with yellow circle (broken). In the top control panel, eGFP alone-transfected cell shows a cilium (highlighted with yellow arrows). Images are taken at 60× magnification. (**B**) The quantification of cilia length after *Bbs8* and *Bbs9* shRNA transfection in IMCD3 cells (obtained from **A**). The X and Y axes respectively show transfection category and length (micrometer) of cilia in eGFP transfected cells per seven fields. Data are presented as mean ± SEM, and statistical significance is indicated with p values.

To further validate the contribution of BBS9 to cilia function, we took advantage of an *in vitro* ciliogenesis assay using IMCD3 cells, which normally grow cilia. Knockdown of *Bbs9* using mouse specific shRNA constructs negatively affected ciliogenesis in IMCD3 cells, resulting in more cells with no cilia compared to the control transfection (Fig. 6A, B; Fig. S2). Some of the transfected cells retained their cilia but these were relatively shorter than the controls (Fig. 6B). As BBS9 interacts with BBS8 in biochemical assays [18], knockdown of *Bbs8* also resulted in similar defects in IMCD3 cells (Fig. 6A, B; Fig. S2). The mouse *Bbs9* knockdown data is in concordance with *bbs9*-spMO morphant KV cilia results (see Fig. 5D, E).

## Discussion

Pioneering studies during the last decade have begun to delineate the molecular pathways leading to BBS and other ciliopathies. As BBS patients share similar clinical features, it is believed that BBS proteins function through common molecular pathways. The existence and interdependence of multimeric BBS protein complexes and their influence on ciliogenesis further supports this view. BBS9 is a component of the BBSome complex and reportedly interacts with several BBS proteins [18]. Our studies provide strong evidence in support of the role of *BBS9* in cilia development as its knockdown results in BBS-like syndromic phenotype in zebrafish. An orthologous human BBS9 mRNA rescued the morphant phenotype, but a mutant mRNA (carrying a missense change observed in a BBS9 patient) failed to provide functional complementation, suggesting an evolutionary conservation of BBS9 function.

In humans, a total of seven mutations have been reported in the *BBS9* gene [23]; all are homozygous except one, a compound heterozygote. Our data show the functional conservation of BBS9 protein domain that includes the missense mutation during evolution. However, additional investigations will be necessary to identify the consequence of other known human mutations within the conserved region of zebrafish *bbs9*.

One important question is how the mutations in *BBS9* lead to a syndromic phenotype. BBS9 is required for the assembly of the BBSome [18], which in turn is needed for targeting membrane proteins to the cilium [31]. Hence, loss of (or severely reduced) function of BBS9 could affect the integrity of the BBSome complex and compromise cilia function. Our KV cilia data demonstrate that like many other BBS genes, *Bbs9* is required for cilia development [32]. The defective cilia in KV can affect the left-right laterality [32]. Since we did not analyze heart looping or laterality markers it remains to be tested whether bbs9 morphants have any laterality defects. The retinal degeneration in zebrafish, exhibited by *bbs9*-spMO morphants, is a further indication of cilia dysgenesis similar to the phenotype produced by *Rpgr* knockdown, which causes abnormal ciliary transport [33].

BBS patients suffer varying degrees of cognitive dysfunctions [34], possibly due to dyskinesia [35] and subsequent development of hydrocephalus, observed in BBS3 patients [29] and its rodent models [36]. Similar phenotypes are reported in rodents [28], [37] and in zebrafish [26]. Thus, the hydrocephaly in *bbs9*-spMO morphant could be attributed to ciliary dyskinesia. Hydrocephaly in *bbs9*-spMO morphants suggested a possible ciliary abnormality in the ventricles and ependymal canal. However, we did not analyze the cilia in these structures. *bbs9*-spMO morphants show defects in neural tube closure, which could be due to defective non-canonical Wnt (PCP) pathway mediated via Vangl2 [38]. Mice having mutations in *BBS1*, *BBS4* or *BBS6* reportedly display a phenotype resembling a mutation in *Vangl2*, which includes neural tube defects [30]. Interestingly, VANGL2 and BBS proteins co-localize in the basal body and ciliary axoneme [5]. We therefore propose that *bbs9* knockdown results in ciliary dysfunction in the morphants, resulting in open neural tubes.

Several components of the BBSome are critical for ciliogenesis. The roles of BBS1, BBS5, and BBS8 in ciliogenesis have been demonstrated in RPE cells [18], [39]. BBS9 has been shown to interact with BBS1 and BBS8, with variable strength [18]. Though an earlier ciliogenesis assay using RPE cells showed a weak effect of BBS9 siRNA [39], our assay using IMCD3 cells and BBS9 shRNA conclusively demonstrated that BBS9 is required for the development of cilia. Defects in cilia function can account for abnormalities in eye and brain of *bbs9*-spMO morphants. Notably, an association between an amino acid change in PTHB1 and premature ovarian failure in human has been reported [22]. The exact pathogenic mechanism is unclear; however, ciliary dysfunction has been associated with ovarian function [40].

In summary, we provide *in vivo* evidence of *bbs9* function in cilia biogenesis and/or transport. Loss of BBS9 leads to defects in organogenesis, presumably because of its crucial role in BBSome assembly and cilia formation. Further investigations are necessary to elucidate the precise biochemical role of BBS9 within the BBSome complex and in cilia biogenesis and/or function.

## Materials and Methods

### Morpholino injections in zebrafish

Fluorescein-tagged morpholinos (MOs) were procured from Gene Tools Inc. (OR, USA). A standard negative control (control-MO), *p53*-atgMO (5′- GCGCCATTGCTTTGCAAGAATTG - 3′) and custom-designed translation blocking (*bbs9*-atgMO - 5′-CGCTGAAGCCAGAACTGTGGAACAT - 3′) and splice blocking (*bbs9*–spMO - 5′-CGGTGCCTGAGAAAACCATACATAT - 3′) MOs against zebrafish *bbs9* were obtained in lyophilized form, re-suspended in distilled water, and quantified spectrophotometrically (NanoDrop Tech Inc, DE, USA).

Zebrafish (*Danio rerio*) were maintained under an approved National Institutes of Health animal use protocol. Staged wild type embryos of EK strain between 2–8 cells were microinjected 0.4–1.2 nL of morpholinos into the yolk sac using pneumatic pico pump (WPI, FL, USA). Before injection, a fresh glass capillary needle was pulled with Kopf needle/pipette puller (Model 750, Tujunga, CA, USA), and calibrated against a micrometer to determine the volume delivered per pulse. Microinjected embryos were incubated at 28°C overnight and scored for survival the following day. Live embryos were ascertained of successful injection by fluorescein signal and followed until 48–72 hpf to observe any overt phenotype under Leica Microscopes MZ16F and ICA (Leica Microsystems, IL, USA). Phenotypes were captured with Leica DC500 camera attached to Leica microscope MZ16F (total magnification employed: 0.63××4× or 8×). Eye size was determined by imaging embryos on a Zeiss Axioskop upright microscope using a 10× objective. Total eye area in pixels was quantified in imageJ using the known scaling factor for this objective. p values were obtained using students t-test (two tailed, unpaired).

### mRNA isolation, RT-PCR and verification of *bbs9* sequence

Total RNA was extracted from individual embryos at 72 hpf with Trizol Reagent (Invitrogen, CA, USA). RNA sample (200 ng) was reverse transcribed with random primers using Superscript III (Invitrogen, CA, USA.). The cDNA was then amplified according to standard protocol using Taq polymerase (New England Biolabs, MA, USA). The following PCR primers were used for splice verification:

5′-TTTGTTTAAGGCCCGTGATT-3′ and 5′-TGAAGGAGTCTGTGCGAATG-3′. Exon 1 to 5 were generated by PCR using primers: 5′-ATGTTCCACAGTTCTGGCTTCAG-3′ and 5′-CTGTAACACCACCGAATGGGCCATA-3′, and the PCR product was TA cloned in pGEMTEasy (Promega Corp, WI, USA). The presence of exons 2 to 5 in pGEMTEasy was verified (Fig. S1) by sequencing using T7 primer.

### *In vitro* RNA preparation for rescue and mutation synthesis

For rescue experiment, a human BBS9 full-length cDNA (Clone ID 5519851) was obtained from Open Biosystems (AL, USA) and sequence-verified. To recreate the missense mutation (G141R) in human BBS9 protein, a corresponding wild type zebrafish nucleotide was subjected to site-directed mutagenesis using QuikChange II kit (Stratagene; Agilent Tech, CA, USA). The wild type and mutant cDNA from SPORT6 were subcloned into pcDNA3.1(+) vector at EcoRV-XhoI sites. Subcloned cDNAs were linearized with XmaI and used for *in vitro* synthesis of capped mRNA using mMESSAGE mMACHINE T7 Ultra Kit (Ambion, Applied Biosystems, CA, USA). The quality of *in vitro* synthesized mRNA was checked on Bioanalyser (Agilent Tech, CA, USA) before co-injection with *bbs9*-spMO.

### *In situ* hybridization using zebrafish embryos or larvae

RT-PCR product corresponding to zebrafish *bbs9* was cloned into pGEMT-easy (Promega Corp, WI, USA) and sequence-verified. The vector was linearized with Sal I or Nco I and used to generate the sense (T7 RNA polymerase) or antisense (SP6 RNA polymerase) probes, respectively, with DIG RNA labeling Mix (Roche Applied Science, IN, USA). *In situ* hybridization was performed as described [41].

### H&E staining of eye and brain

At 72 hpf, zebrafish larvae were fixed with 4% glutaraldehyde for 30 min at RT, then fixed with 4% paraformaldehyde (PFA) overnight at 4°C. Subsequently, they were washed with PBS and embedded in OCT compound Tissue-Tek (SakuraFinetek USA, Inc, CA, USA) and 10 µm sections were cut. The sections were stained with standard H&E staining protocol.

### Staining of Kupffer's vesicle cilia

The embryos aged 10–13 hpf were fixed with 4% PFA and stained with antibodies against acetylated-alpha- tubulin and gamma-tubulin to visualize the cilia. The embryos were then embedded in 2% low melting agarose and positioned for confocal microscopy. The images were taken with Leica TCS SP2 using a water immersion lens (40×) and processed for maximum projection and quantification of cilia using LCM software.

### Ciliogenesis assay

Adult mouse kidney Inner Medullary Collecting Duct cells -3 (IMCD3; ATCC Number: CRL-2123; ATCC, VA, USA.) were grown near confluence overnight on 6-well plate by seeding 200×10∧3 cells and transfected with 750 ng of each plasmid DNA (eGFP and shRNA) in serum free medium using fugene6 (Roche Applied Science, IN, USA). After 8 hr, the serum free medium was replaced with complete medium. Twelve hr later, the cells were washed twice for 5 min each with 0.5 mL PBS and fixed for 15 min at RT with 0.5 mL of 4% PFA in PBS. After fixation, PFA was removed and the cells were washed twice with 0.5 mL PBS. Subsequently, the cells were incubated at RT in 0.5 mL of 5% normal goat serum in PBT (0.1%) for 30 min for blocking. The cells were then incubated with 0.2 mL of a primary antibody (anti-acetylated alpha-tubulin, Sigma -T7451 and anti-gamma-tubulin, Sigma-T6557 (Sigma-Aldrich Corp., MO, USA), both 1∶1000 diluted in blocking solution) for 1 hr at RT. Subsequently, the cells were washed 3× with 0.1% PBT for 5 min each, and incubated with 0.2 mL secondary antibody (anti-mouse Alexa 568 (Invitrogen, CA, USA), 1∶500 diluted in blocking solution) for 1 hr at RT. Finally, nuclear staining was performed with 0.2 mL DAPI (diluted 1∶1000) for 5 min at RT. The cells were washed with 0.5 mL PBS twice for 5 min. The slides were mounted with flouromount and imaged on Olympus FluoView FV1000 (Tokyo, Japan) confocal microscope.

shRNA construct sets against mouse *Bbs8* and *Bbs9* were obtained from Open Biosystems (AL, USA): (*Bbs8*: TRCN0000113210 - 14), (*Bbs9*: TRCN0000178683; TRCN0000181485; TRCN0000182069; TRCN0000182387; TRCN0000182647). The following shRNA constructs gave the best knockdown results - *Bbs8.3*: TRCN0000113213, and *Bbs9.5* (TRCN0000182387) (shown in Fig. 6). The presence or absence of cilia in a transfected (eGFP alone or co-transfected with shRNA construct) cell was manually scored under an epifluorescence microscope (Olympus, BX50F4; 40×; Olympus, Japan). The raw data for all shRNA constructs are presented in Fig. S2. The cilia length in transfected cells (eGFP, BBS8.3 and BBS9.5) was quantified using ImageJ software.

## Supporting Information

Figure S1 **Validated zebrafish** ***bbs9*** **sequence.** The *bbs9* cDNA sequences are aligned to see the degree of matching (top - t. and the bottom - b. sequences were obtained from sequencing data and the provisional version, respectively. The *bbs9* specific product was amplified by PCR using cDNA generated from zebrafish total mRNA. RT-PCR and sequencing data show that exons 2 to 5 are expressed in zebrafish. Exons 2 and 5 are highlighted in yellow; the sequences are perfectly matched until exon 5 (indicated by red bar on top).
(TIF)

Figure S2 ***Bbs8*** **and** ***Bbs9*** **knockdown compromised ciliogenesis in IMCD3 cells.** Knockdown of *Bbs8* and *Bbs9* in IMCD3 cells with 4 different shRNA constructs (#1, 3, 4, 5). Green cells represent the cells transfected with shRNA construct. X-axis displays the analysis categories. Y-axis displays the number of green cells. BBS8 or BBS9 shRNA construct (as indicated) was used along with eGFP. Control transfection was performed with eGFP (shown on the right) without any shRNA construct. Only green cells were counted for obtaining the raw data.
(TIF)
