# Supplementary figures and images for: Knockdown of Bardet-Biedl Syndrome Gene BBS9/PTHB1 Leads to Cilia Defects
Source: PLoS One. 2012 Mar 29;7(3):e34389. doi: 10.1371/journal.pone.0034389 (PMC3315532; doi:10.1371/journal.pone.0034389)

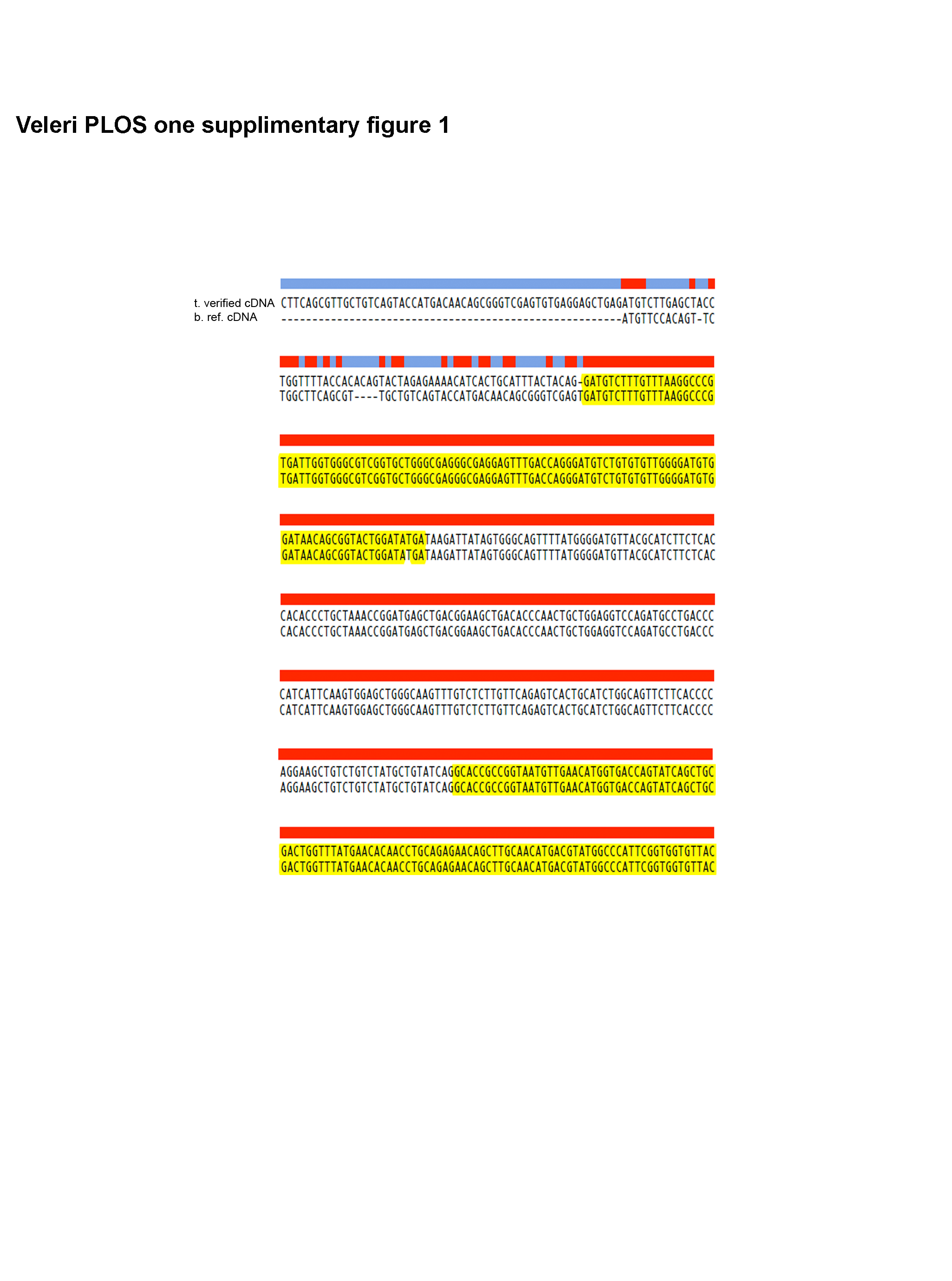

Supplement: Figure S1 — Validated zebrafish bbs9 sequence. The bbs9 cDNA sequences are aligned to see the degree of matching (top - t. and the bottom - b. sequences were obtained from sequencing data and the provisional version, respectively. The bbs9 specific product was amplified by PCR using cDNA generated from zebrafish total mRNA. RT-PCR and sequencing data show that exons 2 to 5 are expressed in zebrafish. Exons 2 and 5 are highlighted in yellow; the sequences are perfectly matched until exon 5 (indicated by red bar on top). (TIF) [file pone.0034389.s001.tif]

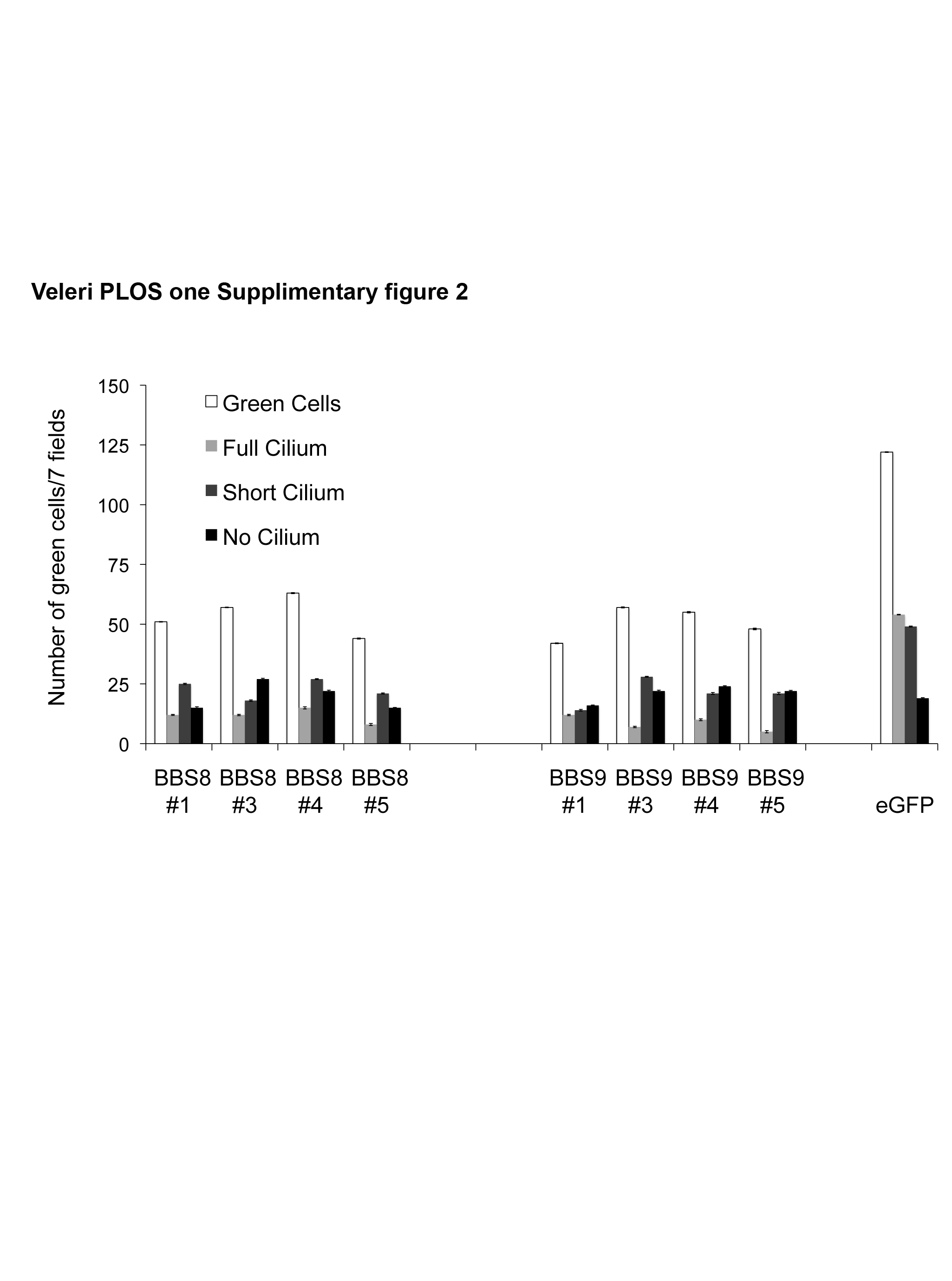

Supplement: Figure S2 — Bbs8 and Bbs9 knockdown compromised ciliogenesis in IMCD3 cells. Knockdown of Bbs8 and Bbs9 in IMCD3 cells with 4 different shRNA constructs (#1, 3, 4, 5). Green cells represent the cells transfected with shRNA construct. X-axis displays the analysis categories. Y-axis displays the number of green cells. BBS8 or BBS9 shRNA construct (as indicated) was used along with eGFP. Control transfection was performed with eGFP (shown on the right) without any shRNA construct. Only green cells were counted for obtaining the raw data. (TIF) [file pone.0034389.s002.tif]
